# Supplementary material for: Burst-and-coast swimmers optimize gait by adapting unique intrinsic cycle
Source: Commun Biol. 2021 Jan 14;4:40. doi: 10.1038/s42003-020-01521-z (PMC7809443; doi:10.1038/s42003-020-01521-z)
Supplement: Supplementary file 2 — Supplementary informations [file 42003_2020_1521_MOESM2_ESM.pdf]

Supplementary Information

Burst-and-coast swimmers optimize gait by adapting unique intrinsic cycle

G. Li, I. Ashraf, B. François, D. Kolomenskiy, F. Lechenault, R. Godoy-Diana, and B. Thiria

Contents

Part 1 Statistical Analyses 2

1.1 Source data points for figure 2 2
1.2 Linear regression model 4
1.3 Linear mixed-effects model 5
1.4 Box plots, ANOVA and pairwise t-tests 6
1.5 Piecewise linear mixed-effects models 7

Part 2 Numerical Approach 9

2.1. Overview of the numerical approach 9
2.2 Computational fluid dynamic model of a free-swimming fish 13
2.3 Results of simulations 17

Part 1 Statistical Analyses

Statistical inference has been used to make judgements about the variation of T_{bout}, F_i, $\bar{A}$ and DC with U in the experiment. The dataset consisted of the statistical averages of those 4 dependent variables, one value for each individual for each speed U. These averages are those presented in Figure 2 of the paper. The original data points for each fish are plotted in section 1.1 below. The numerical values shown below assume that T_{bout} is in seconds, F_i is in Hertz, $\bar{A}$ and DC are dimensionless and U is in BL/s. Linear regression, linear mixed-model analyses, ANOVA and pairwise t-tests have been applied. All statistical calculations were performed in R (version 3.4.4). To make inference, we set the threshold of 0.05 for significance.

1.1 Source data points for figure 2

In the following figures S1-S4 the source data points for each fish presented in figure 2 are shown.

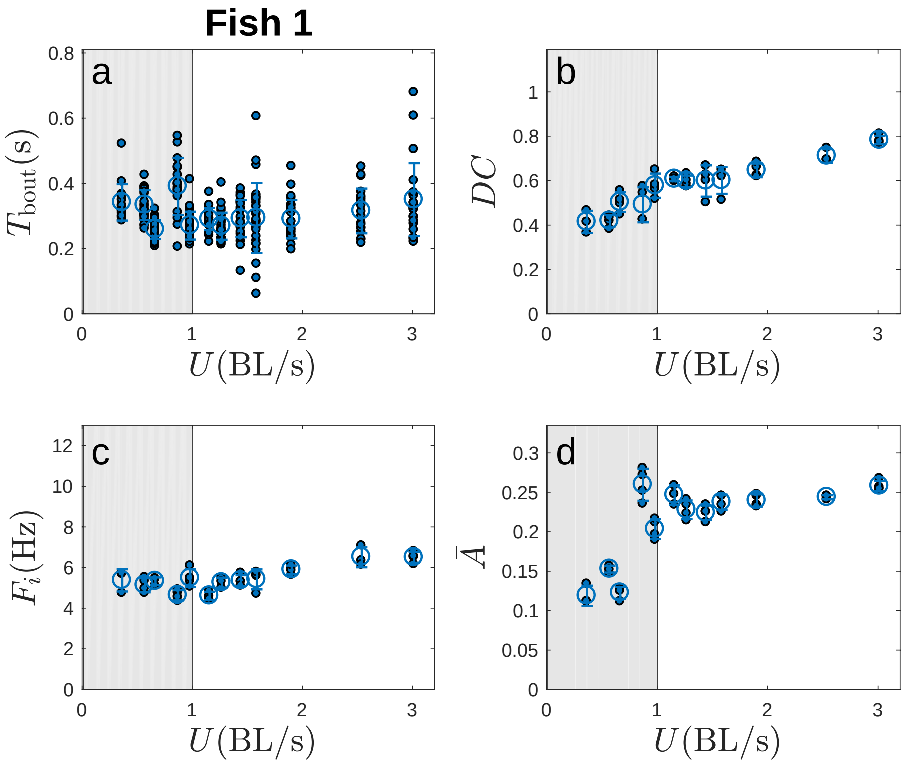

Figure S1. Data points for fish 1 (small circles) and their corresponding averages (big circles) with error bars showing the standard deviation for each velocity.

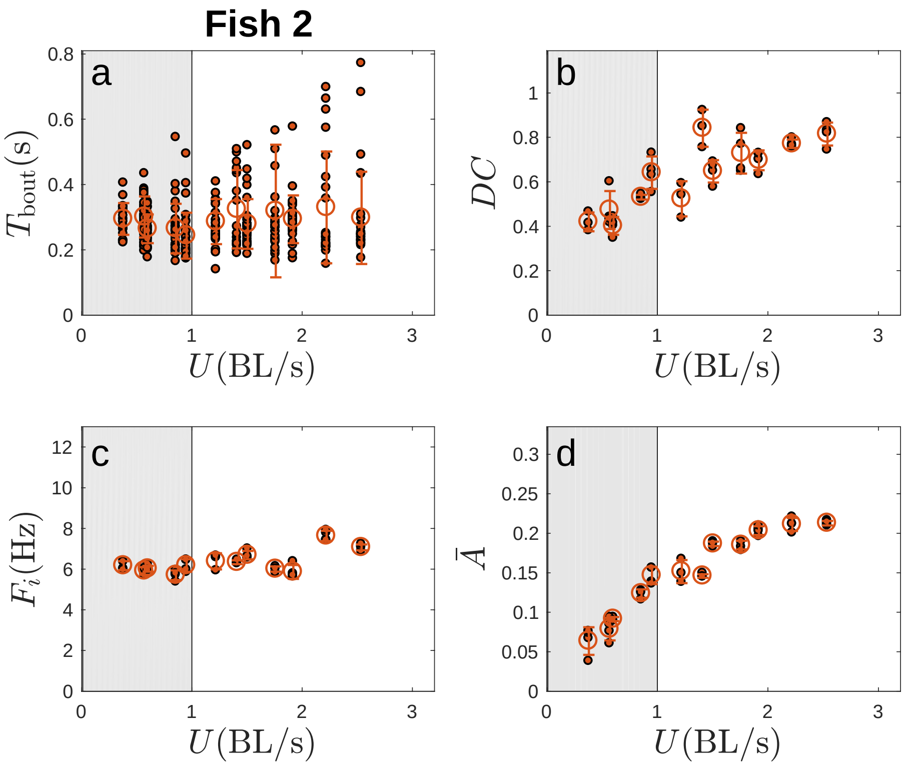

Figure S2. Data points for fish 2 (small circles) and their corresponding averages (big circles) with error bars showing the standard deviation for each velocity.

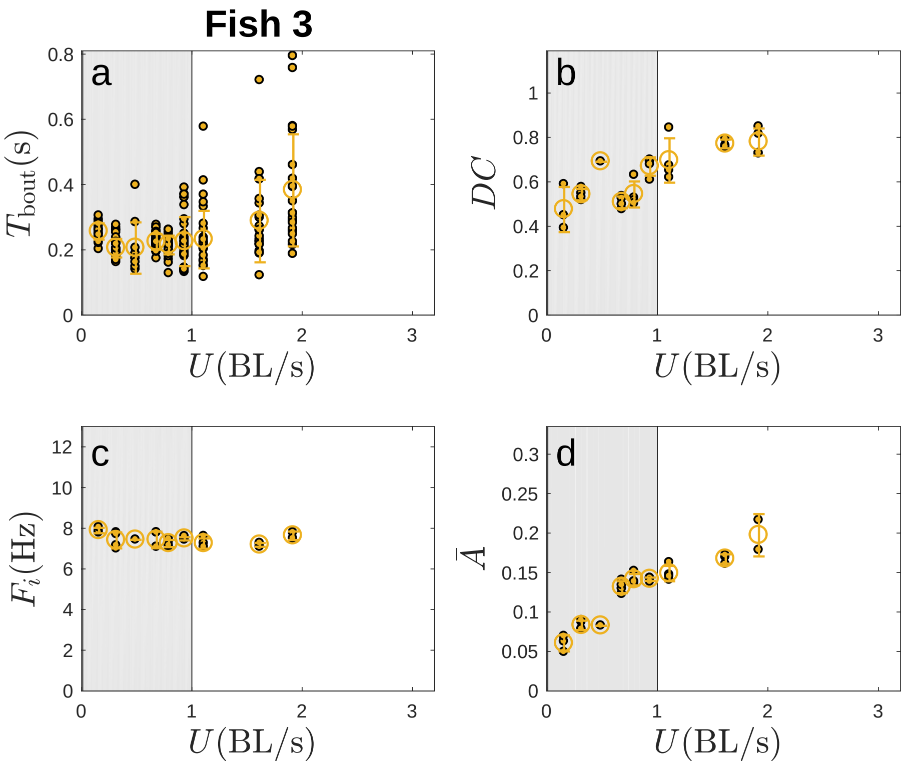

Figure S3. Data points for fish 3 (small circles) and their corresponding averages (big circles) with error bars showing the standard deviation for each velocity.

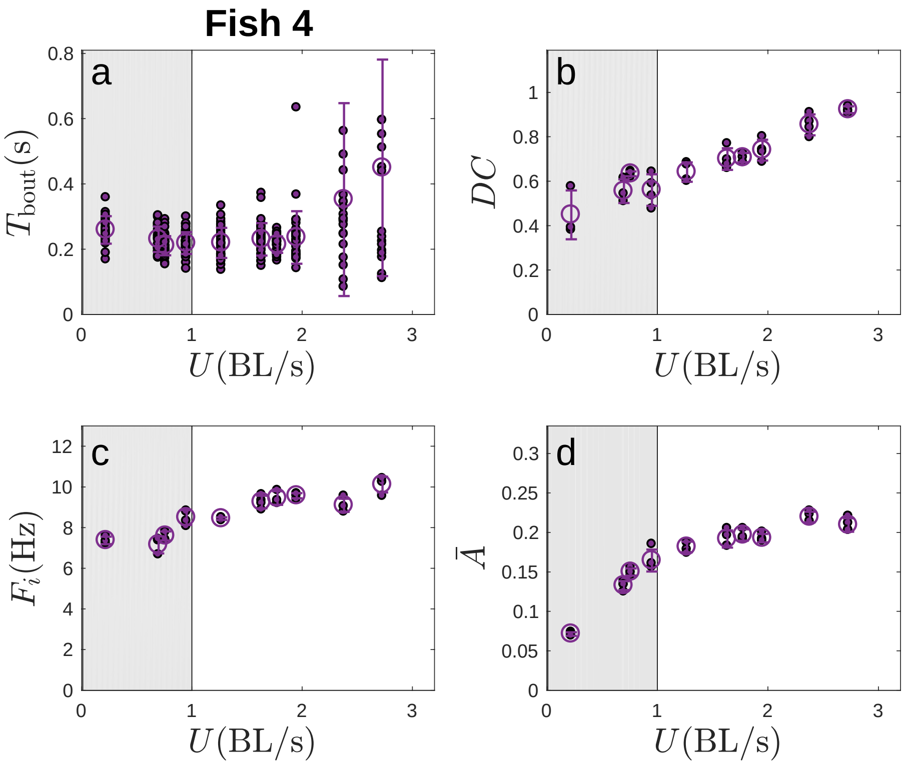

Figure S4. Data points for fish 4 (small circles) and their corresponding averages (big circles) with error bars showing the standard deviation for each velocity.

1.2 Linear regression model

The most obvious trends are quantified using simple linear regression analysis. Its results are summarized in Table S1. For T_{bout} and F_i, simple linear regression accounts for only 25% and 5% of the variability, respectively. Correlation is significant for T_{bout} and insignificant for F_i. For $\bar{A}$ and DC, the linear trend accounts for 70% of the variability, and it is statistically significant.

Table S1. Simple linear regression fit

**Variable**	**Intercept**	**Slope**	**Adjusted R^2**	**Slope p-value**
T_{bout}	0.23298	0.03954	0.2522	0.000356
F_i	6.2194	0.4731	0.04844	0.0839
$\bar{A}$	0.089399	0.058716	0.673	1.01e-11
DC	0.43449	0.14984	0.7095	8.754e-13

1.3 Linear mixed-effects model

Part of the variation that the simple linear regression cannot account for is due to inter-individual variability. Linear trends for a typical individual fish are obtained using a linear mixed-effects model that includes variation across individual fishes as a random effect. We used the *lme4* package. Significance of the fixed effects was tested using likelihood ratio tests (LRT) by fitting nested models (with zero slope) and applying the chi-square test. In addition, profile 95% confidence intervals have been calculated. The results are shown in Table S2. As expected, the fixed effect slopes of the mixed models are slightly different from the slopes obtained from simple linear regression.

For T_{bout} and F_i, simple linear regression accounts for only 25% and 5% of the variability, respectively. Correlation is significant for T_{bout} and insignificant for F_i. For $\bar{A}$ and DC, the linear trend accounts for 70% of the variability, and it is statistically significant. The largest relative difference between those two estimates (10%) is for the slope of F_i. However, for F_i, both models agree on the statistical insignificance of the slope. In particular, in the mixed-effects model, the LRT p-value is 0.06847 and the profile 95% confidence interval for the slope includes zero. Fixed effect slopes for T_{bout}, $\bar{A}$ and DC are significant, and they are almost the same as in the simple linear regression model (less than 9% difference).

Table S2. Linear mixed-effects model fit

Variable	Fixed effect intercept	Fixed effect slope	LRT p-value	Slope 95% confidence interval
T_{bout}	0.23050	0.04126	0.0364	[0.004082856 0.07978224]
F_i	6.2107	0.5192	0.06847	[-0.06144532 1.0890222]
$\bar{A}$	0.090753	0.057152	8.175e-05	[0.04581392 0.06875810]
DC	0.42479	0.16240	8.524e-05	[0.13303696 0.19543660]

1.4 Box plots, ANOVA and pairwise t-tests

In an alternative approach, samples were pooled into three groups: "U1" for the speed less than 1 BL/s (low speed), "U2" between 1 and 2 BL/s (medium speed) and "U4" for the speed greater than 2 BL/s and less than 4 BL/s (high speed). Box plots in Fig. S5 provide a condensed visual representation of the data variation. Then, one-way ANOVA test with no assumption of equal variances and pairwise t-tests with no assumption of equal variances were applied, see the results in Table S3.

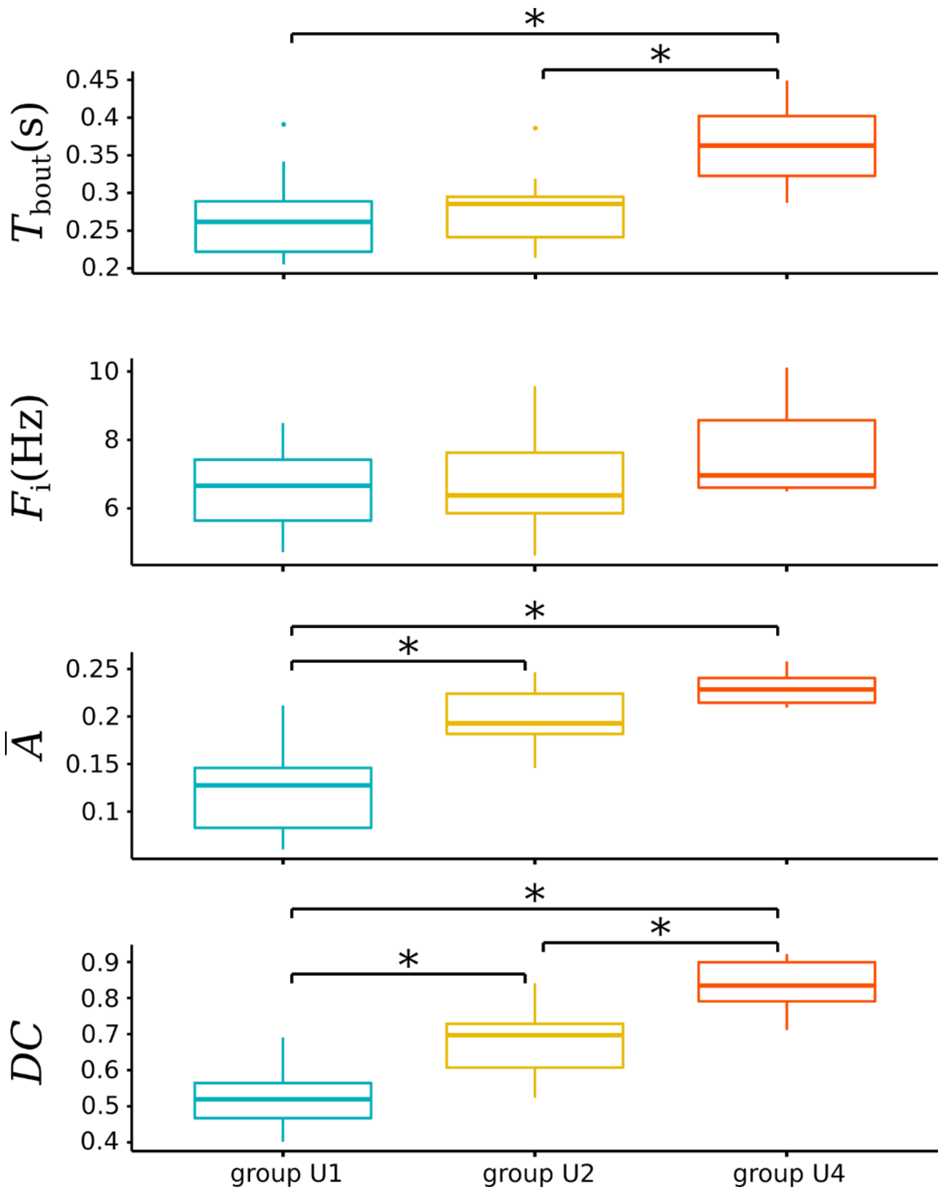

Figure S5. Box plots of T_{bout}, F_i, $\bar{A}$ and DC per group. The asterisk marks statistically significant differences (p<0.05).

In terms of T_{bout}, group U4 is significantly different from U1 and U2, while the difference between the latter two is not significant. F_i, does not show any significant differences across all three groups. For $\bar{A}$, differences between the three groups are significant, although for the pair U4-U2 it is approaching non-significance. In terms of *DC*, all three groups differ significantly.

Table S3. Analysis of variances by speed group

Variable	One-way ANOVA p-value	U2-U1 t-test p-value	U4-U1 t-test p-value	U4-U2 t-test p-value
T_{bout}	0.000426	0.3696	0.0003	0.0013
F_i	0.208	0.50	0.24	0.30
$\bar{A}$	2.31e-09	9.9e-08	9.8e-08	0.042
DC	9.5e-10	8.3e-6	3.7e-5	0.0031

1.5 Piecewise linear mixed-effects models

Pairwise t-tests suggest that T_{bout} and $\bar{A}$ may vary non-linearly with U. Then, it is reasonable to apply a piecewise linear approximation. We divided the full range of velocities in two intervals, according to the pairwise t-test results. In each interval, simple linear regression and mixed-effects modelling have been applied. The results are shown in Tables S4 and S5, respectively. For T_{bout}, both models yield non-significant slopes when the speed is constrained to less than 2 BL/s. At higher speeds, there are not enough points to fit the mixed model for T_{bout}, while the simple regression is applicable but inaccurate (R2<0). Similar piecewise fits for $\bar{A}$ yield significant slopes over the low speed (<1 BL/s) and the medium-high speed (>1 BL/s). The low-speed slope is more than 4 times greater than the medium-high-speed slope.

Table S4. Simple linear regression piecewise fit

Variable	Velocity interval	Intercept	Slope	Adjusted R^2	Slope p-value
T_{bout}	[0 2]	0.25489	0.01535	0.001462	0.3119
T_{bout}	[2 4]	0.18115	0.06702	-0.09595	0.495
$\bar{A}$	[0 1]	0.03824	0.12839	0.6744	5.515e-06
$\bar{A}$	[1 4]	0.151919	0.028178	0.2403	0.01023

Table S5. Linear mixed-effects model piecewise fit

Variable	Velocity interval	Fixed effect intercept	Fixed effect slope	LRT p-value	Slope 95% confidence interval
T_{bout}	[0 2]	0.25986	0.01101	0.5887	[-0.040123739 0.06132525]
T_{bout}	[2 4]	N/A	N/A	N/A	N/A
$\bar{A}$	[0 1]	0.04083	0.12519	0.0001866	[0.095904551 0.15584331]
$\bar{A}$	[1 4]	0.143242	0.031129	0.007688	[0.015460619 0.04726654]

Part 2 Numerical Approach

2.1. Overview of the numerical approach

We developed a numerical model that can generate arbitrary burst-and-coast swimming gait in a four-dimensional parameter space. The parameters are: 1) frequency of the burst phase f_b, 2) amplitude of the burst phase A_b, 3) upper speed bound U_U (the speed at which fish stops burst and start coast) and 4) lower speed bound U_L (the speed at which fish stops coast and start burst, $U_L < U_U$). Then, we search across this parameter space for an optimal burst-and-coast swimming gait that would guarantee sustained swimming with some specified speed $\overline{U}$ at the lowest cost of transport. Numerical solution of this constrained optimization problem involves a coarse discretization of the parameter space, composition of a data base of different gaits with those few discrete values of the frequency and amplitude, and subsequent interpolation using that data base.

The following Fig. S6 explains the details of the numerical approach.

Kinematics & Parameters

The instantaneous body shape is driven by sinusoidal variation of the midline:

$$H(l,t) = \alpha \cdot l^2 \cdot \sin\left(\frac{2\pi l}{\lambda} - 2\pi f t\right)$$

where l is the dimensionless distance from the snout along the longitudinal axis of the fish based on the length of the fish model L;$H(l,t)$ is the dimensionless lateral excursion at time t;α is the dimensionless amplitude envelope function at l; $\lambda = 1.1L$ is the length of the body wave;f is the tail beat frequency. This equation may cause total body length along the midline to vary during the tail beat, which is corrected by a procedure that preserves the lateral excursion$H(l,t)$ while ensuring that the body length remains constant.

We simulated 25 full burst-process simulations, with 5 different tail beat frequencies $f = 2$, 6, 10, 14, 18Hz, and 5 different amplitude control factor $\alpha = 0.02, 0.06, 0.10, 0.14$ & 0.18.

Simulation of a full burst-process

In each free-swimming simulation, the fish accelerates from static condition and until reaching stable terminal speed. The time sequences of speed and power are recorded. The speed and power time sequences were smoothed to remove the periodic fluctuation caused by discontinuous tail beat.

To make the optimization procedure simpler, the fluctuation in speed and power is smoothed by period-averaging.

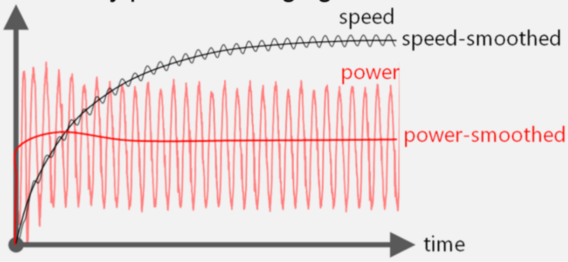

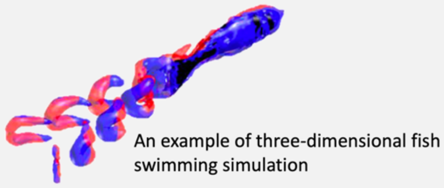

To obtain an arbitrary burst process by interpolation

Each combination of tail beat frequency and amplitude determines a full burst-process, which includes time sequences of speed and power. Based on these 25 sample simulations, for an arbitrary combination of tail beat frequency and amplitude, its corresponding full burst-process (time sequences of speed and power) is obtained by interpolation. *Note that the interpolation result is not a value, but time sequences of speed and power.*

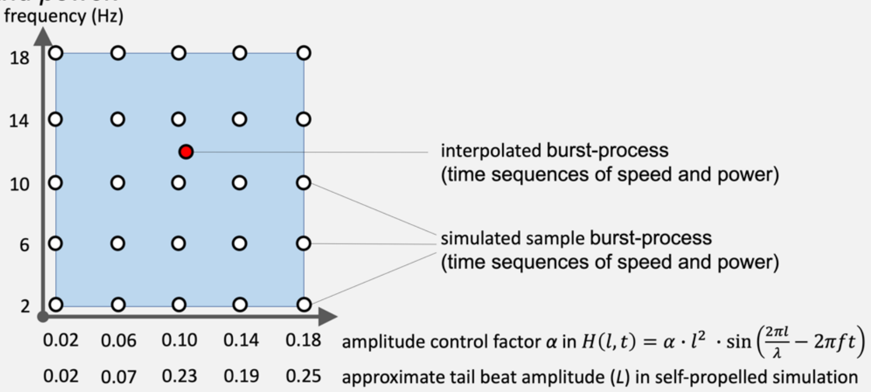

Figure S6. Numerical approach.

To obtain the full coast process

We assume that when fish keep its body straight, the drag of fish only depends on its instant coast speed, and the instant power output is zero. Therefore, we implemented one single coast swimming simulation of which the speed covers entire speed range of all burst simulations (started above 13L/s and ended at almost zero).

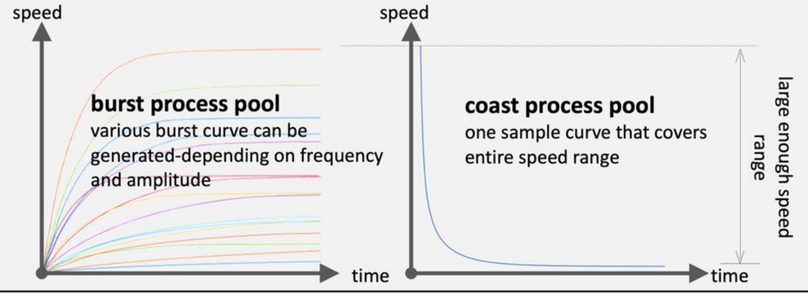

Burst-and-coast gait assembly

There are 4 parameters to define a burst-coast gait: 1) frequency f of the burst phase, 2) amplitude factor α of the burst phase, 3) upper speed bound U_U, and 4) lower speed bound U_L. Once a set of parameters is provided (concerning how a set of parameters is provided, see following panels), frequency and amplitude of the burst phase will define a full burst process. Then, U_U and U_L may cut off a block respectively from burst speed curve, burst power curve, coast speed curve and coast power curve. Based on the assumption that the transitions of burst-to-coast and coast-to-burst are instant, burst-and-coast gait can be obtained by assembling burst- and coast- blocks in a staggered pattern.

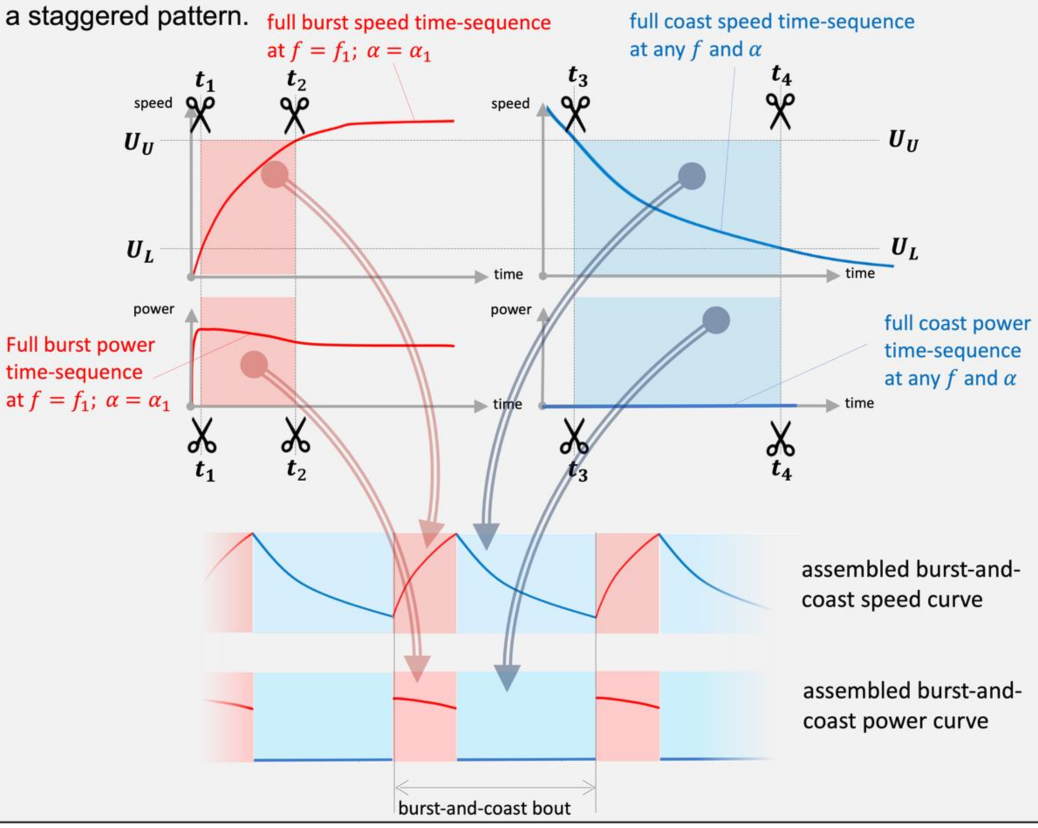

Figure S6 Numerical approach (continued).

Optimization criterion

Based on the assembly of burst-and-coast gait, the following parameters can be defined:

bout duration $T_{bout} = t_2 - t_1 + t_4 - t_3$;

burst duration ratio $\bar{T}_b = \frac{T_{burst}}{T_{bout}} = \frac{t_2 - t_1}{T_{bout}}$;

burst-and-coast bout forward distance $S = \int_{t_1}^{t_2} U_{burst}(t)dt + \int_{t_3}^{t_4} U_{coast}(t)dt$;

burst-and-coast bout energy consumption $E = \int_{t_1}^{t_2} P_{burst}(t)dt + \int_{t_3}^{t_4} P_{coast}(t)dt$;

burst-and-coast bout average speed $\bar{U} = \frac{S}{T_{bout}}$;

burst-and-coast bout average cost of transport $CoT = \frac{E}{S} = \frac{\int_{t_1}^{t_2} P_{burst}(t)dt + \int_{t_3}^{t_4} P_{coast}(t)dt}{\int_{t_1}^{t_2} U_{burst}(t)dt + \int_{t_3}^{t_4} U_{coast}(t)dt}$;

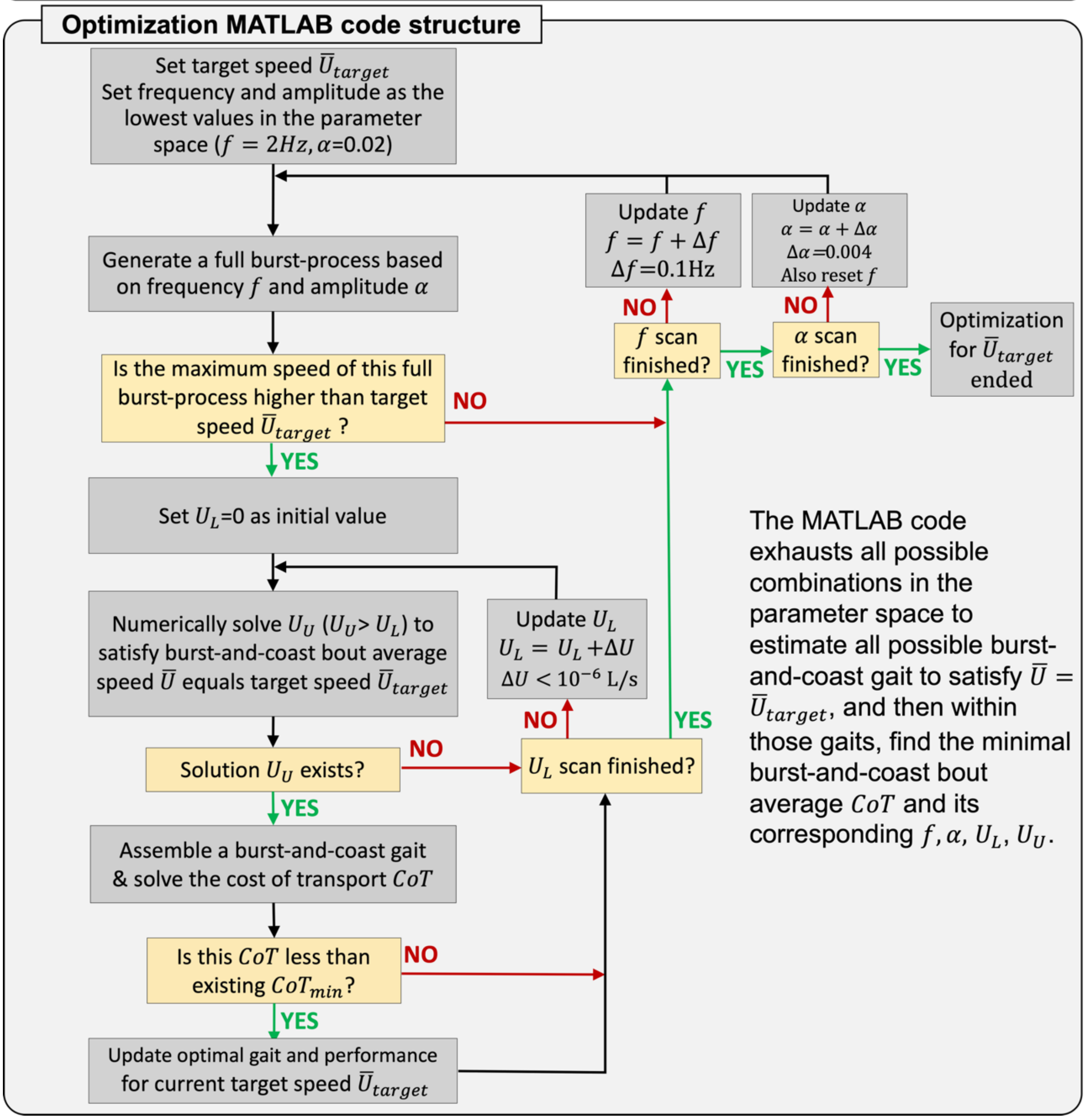

Figure S6 Numerical approach (continued).

2.2 Computational fluid dynamic model of a free-swimming fish

Overview and validations

To obtain the full burst processes and the full coast processes, an in-house three-dimensional computational approach was developed to simulate cyclic swimming of fish (previously described in Li, et al., 2012, 2014, 2016). The model fish swims freely in the horizontal plane (3 degrees of freedom (DoF)). The centre-of-mass (CoM) movements and body orientation are not prescribed, but are determined by the hydrodynamic and inertial forces generated by the swimming model fish. The forces acting on the body and motion of the body are obtained through coupling the hydrodynamic and body dynamic solutions, which ensures that the motions of the fish correspond to the hydrodynamic and inertial forces exerted on the fish.

Locations of detail methods and validations are list in Table S6.

Table S6. Locations of the specific information regarding the computational approach.

specific information		where to find
methods	equations of hydrodynamic solution	Liu, 2009
	equations of motion solution	Li, et al., 2014, 2016 (in supplementary materials)
	coupling of hydrodynamic and motion solution	Li, et al., 2012, 2014, 2016
	multi-grid system and inter-grid communication	Liu, 2009; Li, et al., 2012
	body deformation control	Li, et al., 2012
	inter-body cell	Li, et al., 2016 (in supplementary materials)
validations	grid resolution independence test	Li, et al., 2014 (in supplementary materials)<br>Li, et al., 2019 (in supplementary materials)
	grid size independence test	Li, et al., 2016 (in supplementary materials)
	validation on hydrodynamic solution on oscillating cylinder, compared with experiment	Li, et al., 2014 (in supplementary materials)
	validation on hydrodynamic solution on fixed cylinder, compared with experiment	Li, et al., 2016 (in supplementary materials)
	validation on flow field on swimming fish, compared with PIV	Li, et al., 2012;<br>Li, et al., 2016
	validation on motion solution on swimming fish, compared with experiment	Li, et al., 2012;<br>Li, et al., 2014

Computational grids and fish model deformation

As shown in Fig. S7, the approach comprises surface models of the changing fish shape (dimension: 121 × 97), and local fine-scale body-fitted grids (dimension: 97×121×20 at Re<1000, 97×121×60 at Re>1000) plus a large stationary global grid (dimension: various) to calculate the flow patterns around the fish with sufficient resolution.

The global grid surrounded the body-fitted grids and covered a sufficiently large domain to enclose the swimming fish and their wake. The ensemble of body-fitted grids and global grid was set up as a multi-blocked, overset-grid system based on a chimera grid scheme (Prewitt, et al., 2000). During the simulation, the body-fitted and global grids share values on their interfaces through inter-grid communication algorithm.

The body was modelled on the silhouette of a Red nose tetra fish (*Hemigrammus bleheri*), with a body length of 2 cm. At this length, the range of the Reynolds number in this study is below 6000, turbulence models are not used, and the grid resolution at *Re*=6000 has been justified in our previous study (Li, 2014). All cross-sections of the fish were modeled as ellipses. To reduce the complexity in modelling and computation, we assume that the hydrodynamic influence of all fins other than the tail fin is relatively minor, and neglect them in the model. Also, for the same reason, the gap of the fork-shaped tail fin is neglected, and the fish model has a triangle-shaped fin instead.

The instantaneous body shape is driven by sinusoidal variation of the midline, cf. (Li, et al., 2016),

$$H(l,t) = \alpha \cdot l^2 \cdot \sin\left(\frac{2\pi l}{\lambda} - 2\pi f t\right) \qquad \text{(Eq. S1)}$$

where l is the dimensionless distance from the snout along the longitudinal axis of the fish based on the length of the fish model L; $H(l,t)$ is the dimensionless lateral excursion at time t;α is the dimensionless amplitude envelope function at l; $\lambda = 1.1L$ is the length of the body wave; f is the tail beat frequency. This equation may cause total body length along the midline to vary during the tail beat, which is corrected by a procedure that preserves the lateral excursion $H(l,t)$ while ensuring that the body length remains constant.

We simulated 25 full burst-process simulations, with 5 different tail beat frequency f = 2, 6, 10, 14, 18Hz, and 5 different amplitude control factor α = 0.02, 0.06, 0.10, 0.14 and 0.18. Note that the amplitude control factor α controls the tail beat amplitude of fish in a reference frame attached to fish head, while the resultant tail beat amplitude in world frame of reference A is solved by simulation. The relation between resultant tail beat amplitude A and tail beat amplitude control factor α is shown in Table S7 and Fig. S6.

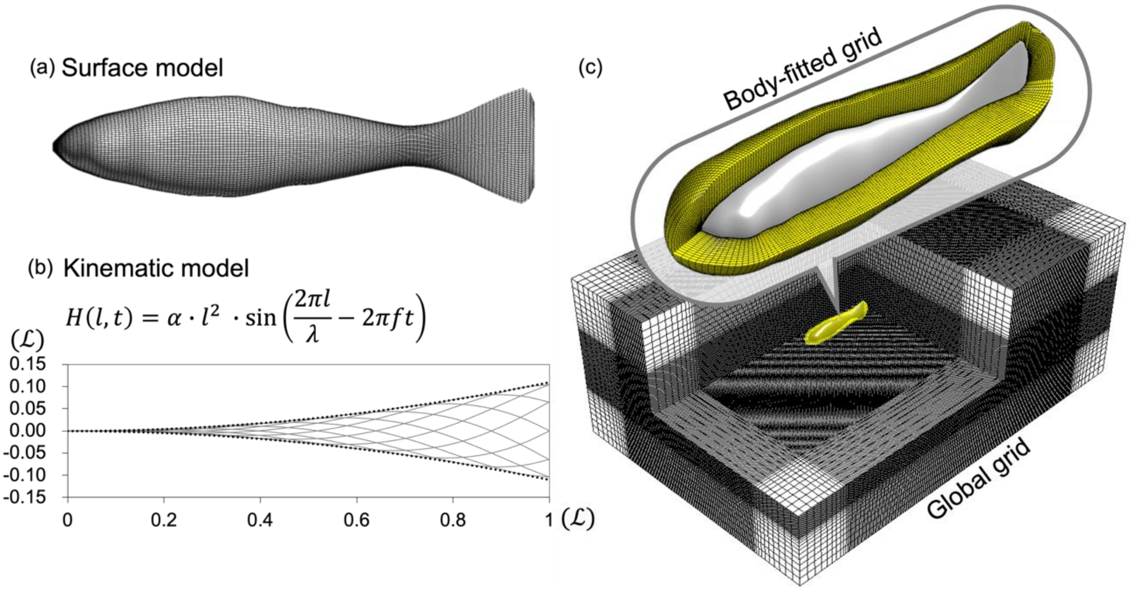

Figure S7. Computational fluid dynamics model. (a) Surface model of a Red nose tetra fish (dimension: 97×121); (b) A function drives the instantaneous body shape. Variation of body length caused by this driving function was corrected to keep lateral excursion and body length constant at 1*L*. (c) Multi-blocked computational grid system consists of local fine-scale body-fitted grid (dimension: 97×121×20 at Re<1000, 97×121×60 at Re>1000) plus a large stationary global grid (dimension: variant).

Body length correction algorithm

Since the sinusoidal functions driving the deformation of fish model may cause total body length along the midline to vary during the tail beat, this variation is corrected by a procedure that preserves the lateral excursion while ensuring that the body length remains constant. Fig. S8 explains the procedure of such correction:

An axis (green axis in Fig. S8) will be firstly generated according to the sinusoidal function. This length of this axis is greater than the original body length. Then, in the longitudinal direction the green axis is linearly contracted until it reaches the original length, while the lateral excursion is preserved. This corrected axis is used as the body shape of the fish in the simulation at this time step.

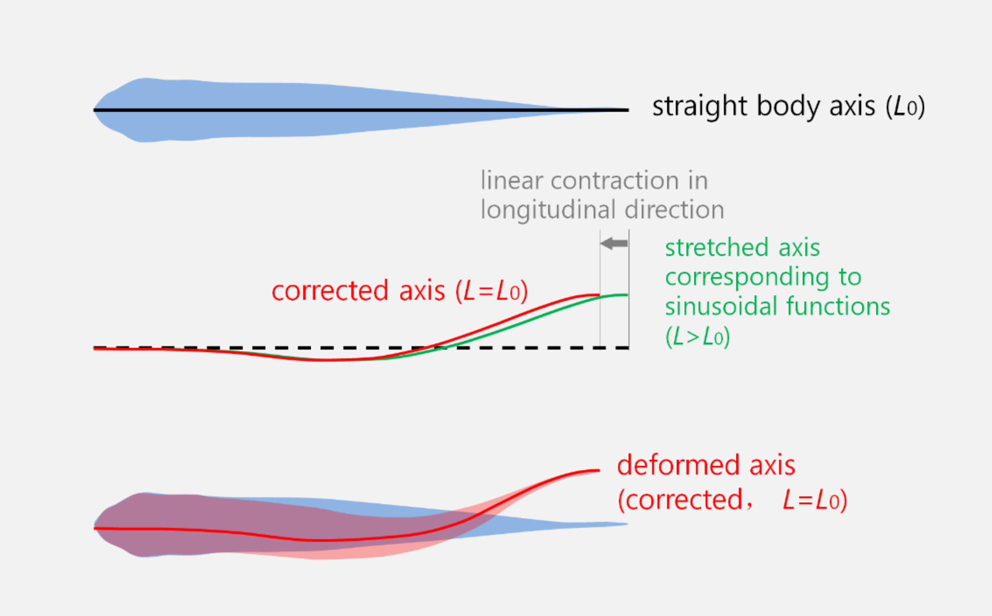

Figure S8. Body length variation caused by deformation is corrected by a procedure that preserves the lateral excursion while ensuring the body length constant

2.3 Results of simulations

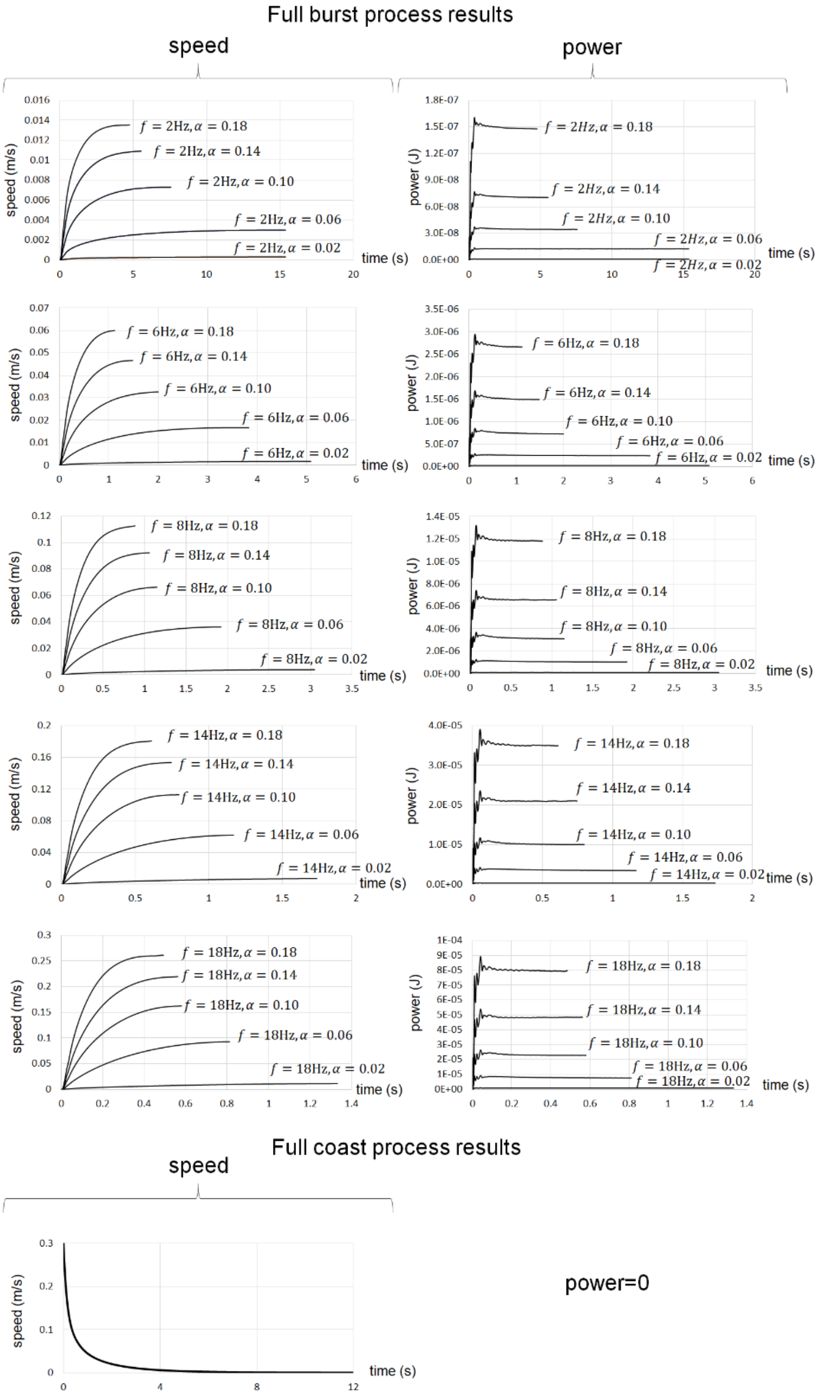

Figure S9. Simulated time sequences of speed and power, respectively in 25 full burst processes and one coast process. Time-sequences of the burst processes were low-pass filtered to remove fluctuations in speed and power caused by undulation. f is the tail beat frequency and α is the tail beat amplitude control factor, the non-linear relation between amplitude A and α is shown in Table S7.

Table S7. The relation between tail beat amplitude A and tail beat amplitude control factor α. Note that A basically depends on α while slightly influenced by tail beat frequency f.

		frequency (Hz)				
		2	6	10	14	18
α	0.02	0.023	0.023	0.023	0.023	0.023
	0.06	0.069	0.070	0.070	0.070	0.071
	0.10	0.118	0.123	0.127	0.131	0.132
	0.14	0.171	0.183	0.190	0.194	0.197
	0.18	0.232	0.244	0.252	0.258	0.263

↑ Amplitude, unit: *L*, 1*L*=0.02m

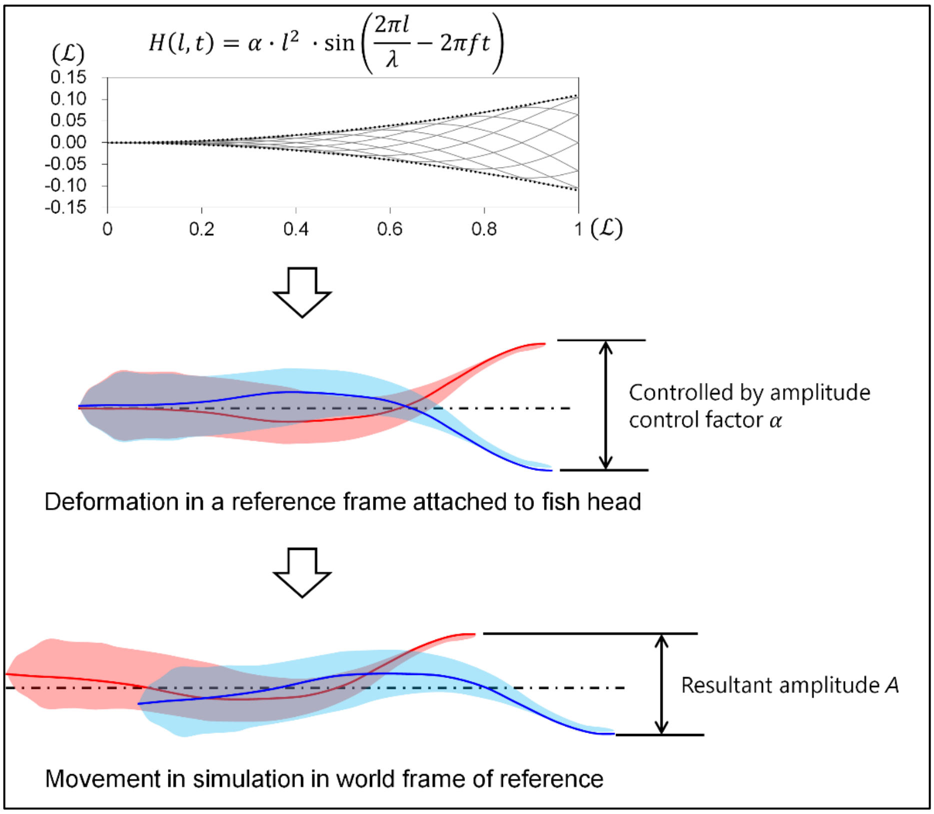

Figure S10. The deformation controlled by tail beat amplitude control factor α, and the tail beat amplitude A measured in simulation. α controls the tail beat amplitude in a reference frame attached to fish head. In self-propelled simulation, the resultant amplitude A is smaller than the tail beat amplitude in the reference frame attached to fish head, since the hydrodynamic recoil and head-swing tend to reduce the tail-beat

amplitude. The relation between tail beat amplitude A and tail beat amplitude control factor α is shown in Table S7.

References

Liu, H. Integrated modeling of insect flight: from morphology, kinematics to aerodynamics. *J. Comput. Phys.* **228**, 439–459 (2009).

Li, G., Müller, U. K., van Leeuwen, J. L. & Liu, H. Body dynamics and hydrodynamics of swimming fish larvae: a computational study. *J. Exp. Biol.* **215**, 4015–4033 (2012).

Li, G., Müller, U. K., van Leeuwen, J. L. & Liu, H. Escape trajectories are deflected when fish larvae intercept their own C-start wake. *J. R. Soc. Interface* **11**, 20140848 (2014).

Li, G., Müller, U. K., van Leeuwen, J. L. & Liu, H. Fish larvae exploit edge vortices along their dorsal and ventral fin folds to propel themselves. *J. R. Soc. Interface* **13**, 20160068 (2016).

Li G, Kolomenskiy D, Liu H, Thiria B, Godoy-Diana R. On the energetics and stability of a minimal fish school. *PLoS ONE* **14**: e0215265 (2019).

Prewitt N. C., Belk D .M., Shyy W. Parallel computing of overset grids for aerodynamic problems with moving objects. Progress in Aerospace Sciences. 2000; 36(2):117–172.
