# Supplementary material for: Burst-and-coast swimmers optimize gait by adapting unique intrinsic cycle
Source: Commun Biol. 2021 Jan 14;4:40. doi: 10.1038/s42003-020-01521-z (PMC7809443; doi:10.1038/s42003-020-01521-z)
Supplement: Supplementary file 7 — Description of Additional Supplementary Files [file 42003_2020_1521_MOESM7_ESM.pdf]

Description of Additional Supplementary Files

File Name: Supplementary Movie 1
Description: (file: Movie_S1_U_0.66BL_s.avi): Video recording of a fish swimming at 0.66 BL/s (body-lengths per second)

File Name: Supplementary Movie 2
Description: (file: Movie_S2_U_1.15BL_s.avi), Video recording of a fish swimming at 1.15 BL/s (body-lengths per second)

File Name: Supplementary Movie 3
Description: (file: Movie_S3_U_1.90BL_s.avi), Video recording of a fish swimming at 1.90 BL/s (body-lengths per second)
